# Comparative Analysis of Detectors and Feature Descriptors for Multispectral Image Matching in Rice Crops

**DOI:** 10.3390/plants10091791

**Published:** 2021-08-28

**Authors:** Manuel G. Forero, Claudia L. Mambuscay, María F. Monroy, Sergio L. Miranda, Dehyro Méndez, Milton Orlando Valencia, Michael Gomez Selvaraj

**Affiliations:** 1 Semillero Lún, Facultad de Ingeniería, Universidad de Ibagué, Ibagué 730002, Colombia; claudia.mambuscay@unibague.edu.co (C.L.M.); 9220191001@estudiantesunibague.edu.co (M.F.M.); sergio.miranda@unibague.edu.co (S.L.M.); 2d20191003@estudiantesunibague.edu.co (D.M.); 2 International Center for Tropical Agriculture (CIAT), Cali 763537, Colombia; m.o.valencia@cgiar.org (M.O.V.); m.selvaraj@cgiar.org (M.G.S.)

**Keywords:** image processing, feature detector, feature descriptor, Brute Force matching, FLANN matching, multispectral images

## Abstract

Precision agriculture has greatly benefited from advances in machine vision and image processing techniques. The use of feature descriptors and detectors allows to find distinctive keypoints in an image and the use of this approach for agronomical applications has become a widespread field of study. By combining near infrared (NIR) images, acquired with a modified Nikon D80 camera, and visible spectrum (VIS) images, acquired with a Nikon D300s, a proper crop identification could be obtained. Still, the use of different sensors brings an image matching challenge due to the difference between cameras and the possible distortions from each imaging technique. The aim of this paper is to compare the performance of several feature descriptors and detectors by comparing near infrared and visual spectral bands in rice crop images. Therefore, a group of 20 different scenes with different cameras and growth stages in a rice crop were evaluated. Thus, red, green, blue (RGB) and L, a, b (CIE L*a*b*) channels were extracted from VIS images in order to compare the matches obtained between each of them and the corresponding NIR image. The BRISK, SURF, SIFT, ORB, KAZE, and AKAZE methods were implemented, which act as descriptors and detectors. Additionally, a combination was made between the FAST algorithm for the detection of keypoints with the BRIEF, BRISK, and FREAK methods for features description. BF and FLANN matching methods were used. The algorithms were implemented in Python using OpenCV library. The green channel presented the highest number of correct matches in all methods. In turn, the method that presented the highest performance both in time and in the number of correct matches was the combination of the FAST feature detector and the BRISK descriptor.

## 1. Introduction

Smart and precision farming can improve crop yield and quality with the ability to predict and prevent diseases, providing flexible and efficient solutions, using unmanned vehicles, drones, and sensors that allow constant monitoring of the crop [1]. Thus, spatio-temporal images acquired with drones can be used to develop statistical models to predict crop yields at different phenological stages [2]. The implementation of sensors to acquire information about the state of crops for monitoring is common in agriculture. Digital, multispectral, and light detecting and ranging (LiDAR) cameras, as well as remote sensors like ground vehicles, unmanned aerial vehicles (UAV) and satellites are commonly employed [3].

The use of visible (VIR) and infrared (NIR) imaging provides information on crop health and growth stage. During the photosynthesis process, chlorophyll molecules of plants absorb blue and red light and reflects green light. On the other hand, infrared light penetrates the inner part of leaves reflecting infrared energy. Since leaf spectral reflectance changes with plants growing, affection of diseases and pest infections, employing these images allows adequate monitoring of crops [4]. Technologies like UAV are implemented to attain information about the crop state in a fast and efficient way. To achieve this task they employ RGB and multispectral cameras. Reflectance bands provide information on leaf structure, chlorophyll content, and nutritional and water stress, which is useful for determining crop health and subsequent yield enhancement [5].

Image processing allows the combination of different sensor techniques, which leads to some challenges. Since the images are taken from two different cameras and at various time periods, rotations and translations between them are unavoidable. In consequence, it is a necessary process to unify acquired data for further analysis. In this case, the image matching process is adequate to detect similarities between them [3]. This process involves the detection and matching of features defined as corners, edges, regions, intensity changes, etc. Feature detection algorithms aim to find these key points. Then, a region is defined around them, from which local descriptors are obtained. Finally, a matching process between comparable features of two or more images is performed [6]. Therefore, imaging techniques are involved in many applications related to object detection [7], pattern recognition, 3D reconstruction, localization strategies, multimodal remote sensing images registration [8], among others. Jiang et al. used convolutional neural networks (CNN) to obtain descriptors and perform classification, using RGB and NIR images as inputs [9].

Additionally, image processing also enables monitoring and estimation of crops yield from vegetation indices [10]. In addition, the use of multispectral images and their processing from feature detectors and descriptors facilitate the inspection of crops at different growth stages [11]. As a result, such images have been widely used in precision agriculture applications. One of the objectives of this approach is to understand the growth and dynamics of crop yields in response to spatial-temporal climate variability and physical environment. This highly facilitate decision-making for farmers [10,12].

There are several comparative studies of image features detectors and descriptors behavior where the aim of the work is to find the most suitable one for images matching. For example, Noble [13] compared the performance of six feature descriptors: invariant feature transform (SIFT), speeded up robust features (SURF), oriented FAST and rotated BRIEF (ORB), binary robust invariant scalable keypoints (BRISK), KAZE, and Accelerated KAZE (AKAZE).

Feature matching was performed by fast library for approximate nearest neighbors (FLANN) and exhaustive search, commonly known as Brute Force (BF), methods on a crop taken by the two cameras at different angles. Rondao et al. [6] benchmarked some of the most used detectors and feature descriptors in the state-of-the-art, and implemented different combinations of detectors and descriptors to improve their performance. SIFT and SURF showed better yielding with fast-Hessian features than using difference of Gaussian as an approximation for Laplacian of Gaussian, used to achieve sensitivity to edges and corners. Additionally, this investigation implemented a long wavelength infrared (LWIR) reflectance band against visual band to identify features repeatable, regardless of the movement of the object in images.

Further studies in the state-of-the-art modified feature detectors and descriptors to improve its performance, integrating georeferenced points taken by UAV and SURF descriptor to automate the acquisition from RGB and hyperspectral images [5].

Several works have been carried out on the analysis of image feature extraction methods using feature descriptors and detectors in agricultural applications. Saleem et al. [3] conducted a comparative study between remotely imaged satellite and UAV imagery over the same field, using different feature points and determining their performance. Yan et al. [7] proposed an object detection approach for agricultural environments. Features were extracted from RGB and mono-stereo images by applying the FAST detector. Then, a matching process was carried out to detect objects in a ground vehicle trajectory. Cowan et al. [14] evaluated the performance of the detectors and feature descriptors available in the OpenCV 3.1 library, AGAST, KAZE, AKAZE, FAST, BRISK, BRIEF, FREAK, SIFT, SURF, and ORB, using UAV imagery for visual tracking purposes. Performance was evaluated in terms of computation time, repeatability, and precision. The results showed that the best performances were achieved by BRISK, FREAK, and ORB mainly. Some drawbacks were the absence of visual results on the images and the very general conclusions. Malekabadi et al. studied the combination of different detectors and feature descriptors using BRISK, FREAK, SURF, MSER, and Harris methods to compare tree canopy images in different color spaces, such as HSV, H, YCbCr, Y, NTSC, and RGB with stereo images.The highest number of key points was obtained by the MSER and SURF detectors, the best performing combinations were SURF–SURF, followed by SURF–FREAK and HARRIS–SURF and the best spaces to implement the combinations were RGB and Y [15].

This paper is focused on a benchmarking of different state-of-the-art feature detectors and descriptors: binary robust invariant scalable keypoints (BRISK), features from accelerated segment test (FAST), speeded up robust features (SURF), scale invariant feature transform (SIFT), oriented FAST and rotated BRIEF (ORB), binary robust independent elementary features (BRIEF), fast retina keypoint (FREAK), KAZE, and AKAZE. Furthermore, two matching methods available in open computer vision (OpenCV) library, in Python language, were used: fast library for approximate nearest neighbors (FLANN) and Brute Force (BF). SIFT is a feature detector and descriptor invariant to image scale and rotation, that employ a 3D spatial histogram of the image gradients for feature description. SURF is a feature detector and descriptor very similar to SIFT, that applies an approximate Gaussian second derivative mask to an image at many scales for feature detection. FAST is a corner detection method that compares pixel intensity to identify key points. BRISK is a detector and descriptor robust to image rotation, based on a scale-space FAST method. BRIEF is a binary feature point descriptor that employs simple tests using intensity difference to describe key points in a pair of images. ORB is the result of using the FAST method to detect key points and the BRIEF detector with some modifications. FREAK is a binary keypoint descriptor which employs a highly structured pattern that mimics the human eyes. KAZE is a feature descriptor that aims to detect and describe 2D image features in a non-linear scale space, using the Hessian matrix. AKAZE is the accelerated version of KAZE, using non-linear scale space based on anisotropic diffusion. The used dataset is a set of visible (VIS) and near-infrared (NIR) spectral images of different cropland and rice stages.

## 2. Materials and Methods

### 2.1. Materials

**Figure 1:**
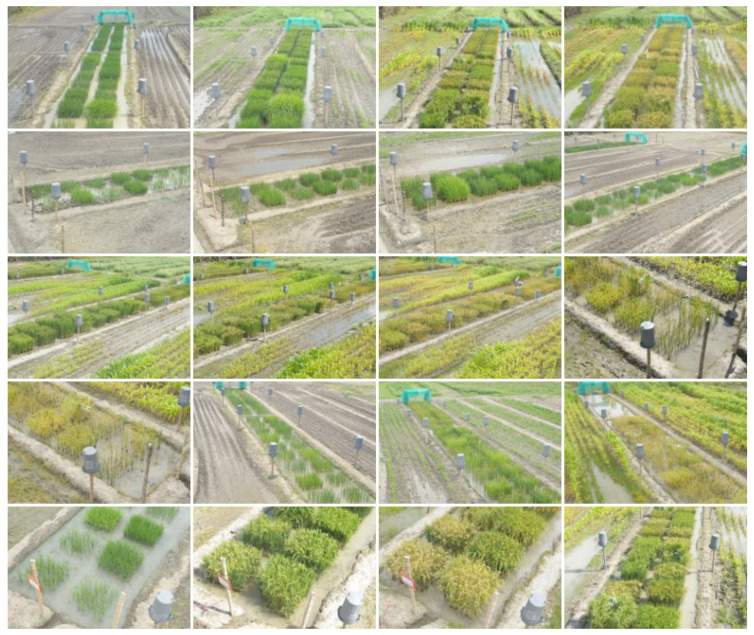
Set of visual (VIS) images of rice crops.

**Figure 2:**
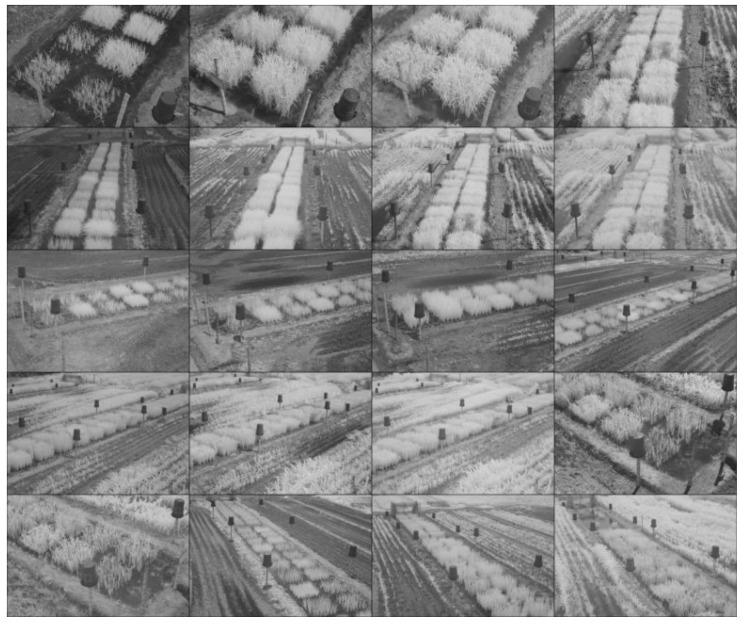
Set of near infrared (NIR) images of rice crops.

A set of 96 images was acquired using two RGB and NIR cameras located on two 8-meter high towers, used as part of a field phenotyping system. In total, 48 RGB images from the visible spectrum were acquired with a Nikon D300s SLR camera and 48 NIR images of the same scenes were acquired with a modified Nikon D80 SLR camera. The latter was modified by placing an IR-85 high-pass filter (HOYA Corp., Tokyo, Japan; transition wavelength > 850 nm) over the CCD sensor to capture in the NIR wavelength regions. For this study, a randomly selected subset of 40 images was taken from the total number of photos, a sufficient number to statistically validate the results obtained. Thus, the dataset consists of two groups of 20 images, corresponding to the NIR and VIS spectra, with sizes of 3872 × 2592 and 4288 × 2848 pixels, respectively. The images were scaled to 774 × 518 pixels to facilitate handling and reduce computational cost. As seen in Figure 1 and Figure 2, the images were taken from different crops, phenological states and angles. The buckets and poles are commonly used as reference elements for the images registration process. The methods were implemented in Python 3.6 language using OpenCV contrib version 3.4.2.16 library. For the VIS images, a channel splitting process was carried out and RGB and CIE L*a*b* images were obtained to analyze the correspondences between each of them and the NIR images based on feature detection and description. The CIE L*a*b* color space, defined by the International Commission on Illumination in 1976, expresses color by the values L* for the perceptual luminance, and a* and b* for the four unique colors of human vision: red, green, blue and yellow. This space is useful for detecting small color differences [16]. The decomposition of the CIE L*a*b* channels was performed using open access software imageJ. The tests were developed in an Intel® Core(TM) i5-8250U CPU @ 1.60 GHz computer with 8GB RAM, running on Windows 10.

### 2.2. Methods

To develop the comparative study, a process of selection of points of interest was initially carried out and then a technique was used to describe them using the algorithms described below.

#### 2.2.1. Evaluated Methods

BRISK, SURF, SIFT, ORB, KAZE, and AKAZE methods were used as features detectors and descriptors. Furthermore, BRIEF, BRISK, and FREAK methods were employed for the features description and FAST algorithm was implemented for keypoint detection. To make a more complete study, the techniques mentioned before, which were developed with the aim of reducing the computational cost of finding matches, were included. For the sake of simplicity, the combination of some detectors and descriptors are named as follows: FAST + BRIEF, FAST + BRISK, and FAST + FREAK. For the FAST + BRIEF method, the threshold used was 25. Meanwhile, for the other approaches, default parameters were employed. These state-of-the-art methods are described below:

- **SIFT:** This method detects the key points by applying a Gaussian filter to smooth the image while rescaling it. In this way, a pyramid is generated with the original image at the first level. The key points are then detected around a 3×3×3 matrix of any pixel at an intermediate level where the Gaussian difference values reach an extreme. The SIFT method is also a key point descriptor, where it performs a histogram of the position of the local image gradient directions around the point of interest being scale invariant [17];
- **SURF:** The keypoint detection process of this algorithm is very similar to SIFT, however, the SURF is based on a Hessian matrix, which is generated by convolution of the Gaussian second-order derivative with image pixels by detecting the keypoints using a 3×3×3 non-maximal suppression in a Gaussian pyramid, and then interpolating the maxima of the Hessian matrix. Keypoint detection is performed by first assigning the orientation by obtaining the Haar Wavelet response in the x and y directions around each point of interest, where the dominant orientation is defined by calculating the sum of orientations. Then, the Wavelet responses in a square region oriented in the dominant orientation provide the SURF descriptors [18];
- **FAST:** This algorithm aims to find points of interest in an image and is specialized in corner detection. To find the points of interest it analyzes each pixel *p*, comparing its intensity with that of the four pixels located in a circle of radius 3, forming a cross with the center *p*. If at least 3 of them are darker or brighter than *p*, *p* is compared with the 16 pixels located on the edge of the circle. If at least 12 of them are darker or brighter than *p*, and are consecutive *p* is identified as a key point [19];
- **BRISK:** Is a detector and descriptor robust to rotational changes, based on a scale-space FAST method introduced by [19]. It takes the input image $c_{0}$ to create a scale space with *n* octaves $c_{i}$ and intra-octaves $d_{i}$, where i={0.1,…,n−1} and normally n=4. To find the points of interest, a circular mask consisting of 16 points located at the edge of the circumference is initially used. If at least 9 of the 16 pixels are larger or smaller than the central one, it is taken as a candidate keypoint. This process is performed in each octave and intra-octave. The candidate points are compared with their neighbors in the same layer and in the upper and lower ones. Thus, points are obtained on different scales and scored accordingly [20]. After obtaining the points of interest at different scales, they are used for the construction of the descriptor. Thus, on these (k) points in the input image a sampling pattern of *n* samples is used, usually (n=60) consisting of four circles. Then, a Gaussian smoothing is performed with standard deviation equal to the distance between the points of the same circle. In addition, the local gradient over the *k* is calculated. Afterwards, two subgroups are defined, the first one formed by pairs of samples separated by a short distance *S* and the other one by pairs separated by a larger distance *L*. In this way, the estimate of the direction of *k* is obtained. For obtaining the rotation invariance, the sampling pattern rotated by $\alpha =arctan2(g_{y},g_{x})$ about the point of interest *k* is used. The description of point *k* is formed by the bit vector $d_{k}$ which is assembled from the short-distance pairwise intensity comparisons. Finally, to compare two BRISK descriptors, the Hamming distance is employed, using the difference between the bits as the dissimilarity measure [20];
- **BRIEF:** Is a binary feature point descriptor that employs simple tests using intensity difference to create binary feature vectors that effectively describe key points in a pair of image patches. Before executing binary tests, images patches are smoothed using a Gaussian kernel at a pixel level, reducing noise sensitivity. The obtained binary strings with BRIEF only require between 128 and 512 bits, a relatively few number of bits compared with other state-of-the-art feature descriptors. The Hamming distance is employed for evaluating the ranking of descriptors, instead of the L2 distance, since it is easier to calculate. Although construction and matching for this descriptor is faster than other state-of-the-art ones, does not provide rotation invariance, but tolerates small amounts of rotations. Since BRIEF does not make features detection, any fast detector, such as CenSurE (Star) or FAST, can be used with this descriptor, but should not be used with SURF as it would negate BRIEF’s speed advantage [21];
- **ORB:** This method results from a fusion between the FAST keypoint detector and the BRIEF descriptor with some modifications. ORB employs the FAST method to detect the key points and implements the Harris corner detector to find the main N points at each level of a multi-scale image pyramid, thus seeking to be invariant to scale change. Orientation invariance is obtained by calculating the intensity centroid of a circular patch centered at the keypoint. The orientation is given by the direction of the vector located between the pixel, located at a corner, and the intensity centroid. ORB uses BRIEF as its feature descriptor doing a ’steer’ BRIEF according to the orientation of the keypoints. To construct the feature set, the result of *n* binary tests at the points of interest *(x,y)* is taken and the orientation of the patch θ around the key point is calculated [22];
- **FREAK:** Is a binary keypoint descriptor which employ a highly structured pattern that mimics the human eyes saccadic search for binary tests. A cascade of binary strings is computed by efficiently comparing pairs of image intensities. The sampling method involves a smoothing process with a Gaussian kernel using a circular retinal sampling grid. The radius of the circular grid represents the size of the kernel standard deviation, corresponding with the distribution of receptive fields over the retina. This descriptor is very similar to BRISK, but unlike this one, an exponential change in size and the overlapping receptive fields in the topology, that captures more information is handled. The added redundancy allows the use of less receptive fields, which is a known strategy employed in compressed sensing (dictionary learning). FREAK also follows ORB’s approach to learn the best pairs from training data by maximizing variance. It takes advantage of this structure to further speed up the matching using a cascade approach: more than 90% of the candidates are discarded with the first 128 bits of the descriptor if the distance is smaller than a threshold. Finally, FREAK compensate rotation changes measuring the orientation in a similar way to BRISK. However, instead of using long distance pairs, a predefined set of 45 symmetric sampling pairs is used [23];
- **KAZE:** The algorithm consists of detecting and describing 2D features in a non-linear scale space to obtain better distinction and location precision. From an input image, a non-linear scale space is constructed by discretizing the scale space in logarithmic steps arranged in a series of octaves and sublevels while maintaining the resolution of the original image. Then, convert the set of discrete scale levels in units of pixels ($\sigma_{i}$) to time units ($t_{i}$). The non-linear scale space is then built iteratively using an additive operator splitting (AOS) and variable conductance diffusion schemes reducing noise and preserving the boundaries of the objects in the image. The 2D feature detection is obtained from the scaled normalized determinant of the Hessian matrix calculated at multiple scale levels, taking the maxima as key points. The description is made from the calculation of the keypoint orientation, obtaining a descriptor invariant to scale and rotation. This technique is computationally expensive [24].
- **AKAZE:** Is an accelerated version of KAZE. It consists of a 2D feature detector and descriptor that uses a faster method to create a non-linear scale space based on anisotropic diffusion called fast explicit diffusion (FED) to create a fine and coarse pyramid frame. In this way, a set of filtered images is obtained to build a non-linear scale space. For feature detection, a Hessian determinant and a modified version of the local difference binary descriptor (M-LDB), which exploits the gradient and intensity information of the non-linear scale space, are used [25].

#### 2.2.2. Evaluation Process

**Figure 3:**
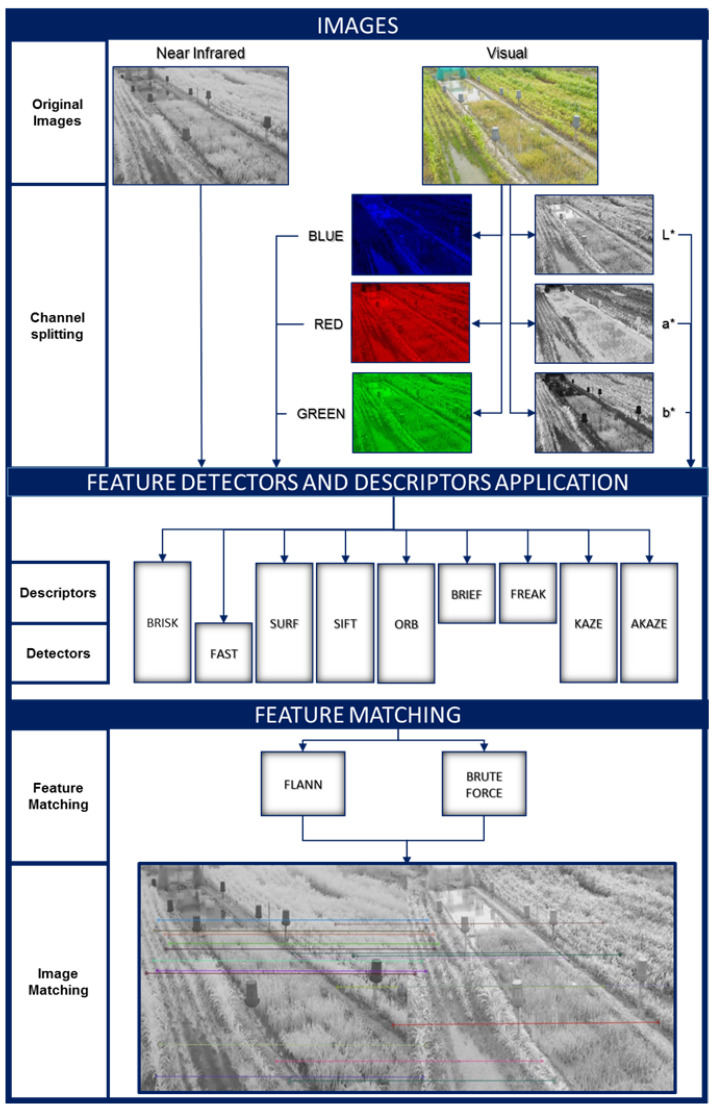
Overview of the feature matching comparison process of feature detectors and descriptors used.

For the evaluation of the points of interest, two color spaces were used: the standard RGB image and one where the intensity information of the image can be isolated in a single channel. The CIE L*a*b* color space was used for this purpose. The implemented methodology is shown in Figure 3. First, the NIR and VIS images were resized to the same dimensions. Then, the VIS images were separated for study into RGB and CIE L*a*b* color space channels. The latter was selected not only because it provides luminance information in a separate channel, but also the variation from one color to another follows a more linear pattern than other color spaces [26]. Subsequently, the feature detectors to be evaluated were used to extract key points from the image. Descriptors were used to generate a vector of features that encode the information around the keypoint. Finally, a matching process was performed between the features of the NIR images and those of the RGB and CIE L*a*b* channels for the same crop at a given growth stage.

The process consisted initially in obtaining the key points and descriptors in the NIR image and in the corresponding image of each channel (red, green, blue, L*, a*, b*). Matching features were then found by Brute Force (BF), using Hamming and Norma L1 distances for matching, as shown in Table 1, and by the FLANN method, using a ratio threshold of 0.7.

**Figure 4:**
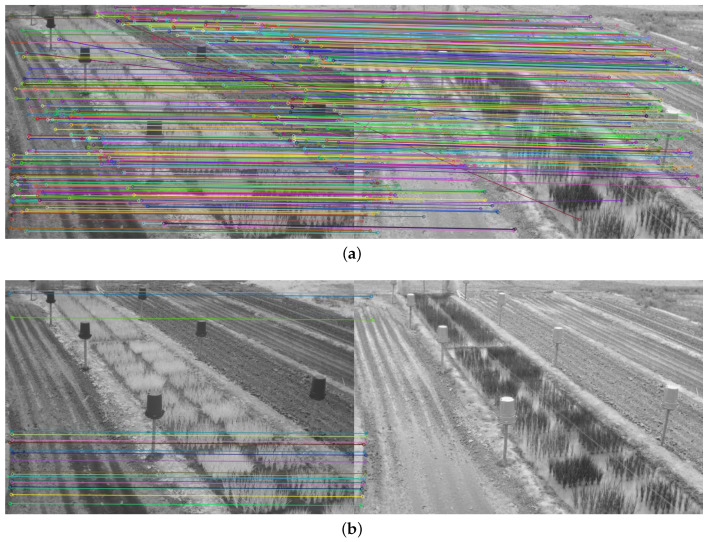
Matching result between a pair of images of the dataset in the NIR and blue band using the detector and the SIFT descriptor, and the FLANN matching method: (**a**) Original matching result; (**b**) Twenty most significant matches.

To determine the performance of the detectors and feature descriptors, the twenty points of interest with the best match between each pair of images were obtained and the execution time measured. The number of correct matches between the twenty points of interest was also observed. Only twenty points were taken, as the number of matches generated from the detectors and feature descriptors was too high (see Figure 4a), which made it difficult to evaluate the quality of the matches, as shown in Figure 4.

The average number of matched keypoints between the NIR image and each color component of the VIS images in RBG and CIE L*a*b* spaces and the average run time were also obtained. The results achieved with the image in Figure 4a) can be found in Appendix A.

## 3. Results and Discussion

Considering the average number of matches found on RGB and CIE L*a*b* images from dataset, the performance of each feature descriptor and detector was evaluated, as it is illustrated in Table 2 and Table 3. Table 2 shows the results using the FLANN matching method. In this method, the highest number of matches was obtained by comparing the NIR images with each component of the RGB space, finding more matches in the green channel. The highest score was achieved using SIFT. On the other hand, the result was very diverse when the correspondence between the NIR images and the CIE L*a*b* space channels was performed, finding the number of coincidences obtained with the L* channel were similar to those with the green channel. In addition, several of the best matching results with the different descriptors and interest point detectors were obtained with the b* channel. Whereas the use of the a* channel yielded poor results in all cases. Table 3 shows the results using BF matching method. In this approach, results were very similar to the previous one. However, the highest score was achieved in the green channel using FAST. Contrasting matching methods, although the difference was relatively small, FLANN achieved the best average match. Nevertheless, in almost all cases, the use of BF matching led to higher average matching values.

**Figure 5:**
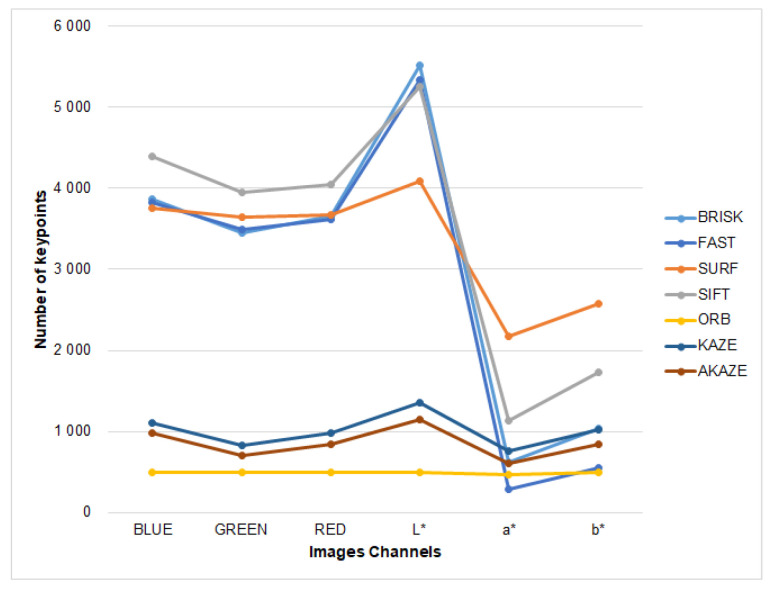
Average number of keypoints found by keypoint detectors evaluated on each channel of RGB and CIE L*a*b* dataset images.

As shown in Figure 5, the highest number of found key points occurred in the L* channel, where the BRISK, FAST, SIFT, and SURF methods detected the highest number of points, between 4000 and 5500 interest points on average. The key points found in the RGB channels were lower than those obtained with the L* channel, while with the a* and b* channels the number was the lowest. The minimum number of points was obtained with the KAZe, AKAZE, and ORB methods, ORB being the technique with which the lowest number was found in all cases. BRISK, FAST, SURF, and SIFT yielded the highest number of keypoints.

**Figure 6:**
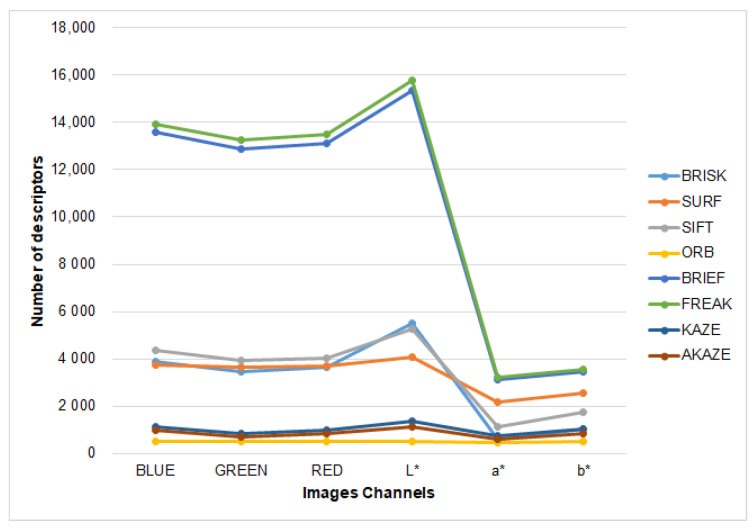
Average number of descriptors found by feature descriptors evaluated on each channel of RGB and CIE L*a*b* dataset images.

In Figure 6 the average number of descriptors detected for each feature descriptor over all channels of images dataset is compared. It is evident that FREAK and BRIEF showed the best performance on finding descriptors. Around 16000 features were obtained on L* channel images. In comparison, ORB, KAZE, and AKAZE found around 500 and 1000 features, and exhibited the worst performance as feature descriptors. On the other side, analyzing Figure 6 results in contrast with Table 2 and Table 3, the use of descriptors with RGB channels showed better feature detection performance than using a* and b* channels.

Execution time was also used to evaluate performance of feature descriptors and detectors, as can be seen in Table 4. As expected, comparing CIE L*a*b* and RGB images, computation time was lower with the first ones for both matching methods, due to the lower number of matches found with those images. The most computationally efficient method was ORB for both FLANN and BF for each matched image pair. On the other hand, FAST + FREAK and KAZE showed the worst time results, using BF and FLANN matching, respectively. Comparing this results with the ones in Table 2 and Table 3, the best performance of ORB method for both time and number of matches was exhibited using BF and CIE L*a*b* images. As expected on literature [6] ORB feature descriptor exhibited the lowest execution time. The diverse results obtained in many cases with FLANN could be due to this one optimised matching search using a nearest neighbour approach. Therefore, multiple parameters can be modified depending on the detector implemented, and a default setting was used. In contrast, BF does not behave in that way because of its simplicity, thus, the results were more homogeneous with this matching method.

The Appendix A shows the matching results obtained for each feature descriptor and detector in an image of the dataset using FLANN and BF matching. Since there was no limit on the matches and the RGB and CIE L*a*b* channels were used, the need for constraints is clear. This is evident in Figure A2 and Figure A4 as a result of the large number of matches found with the BF method.

**Figure 7:**
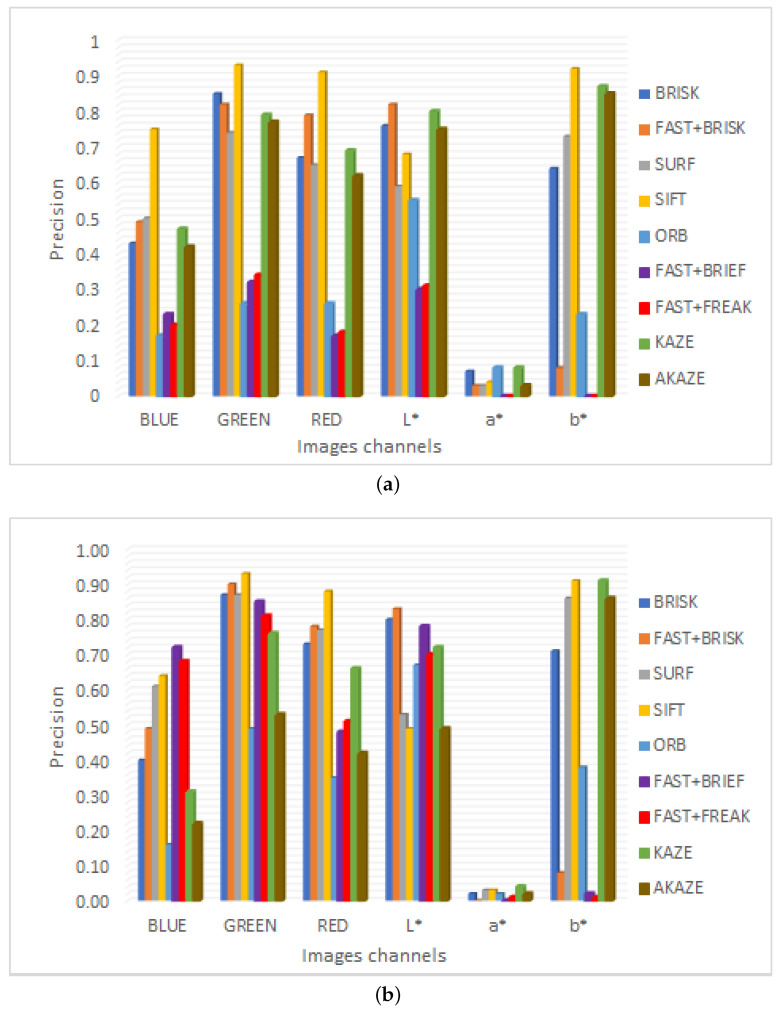
Precision obtained from the first 20 set points. (**a**) FLANN matching; (**b**) BF matching.

To evaluate the performance of the methods, the accuracy of the twenty most significant match points obtained between each pair of images with the different techniques was also obtained. The precision is given by the number of correct matches over the total number of matches [15]. This measure gives a good idea of the quality of the matches obtained with each technique, as shown in Figure 7, where it is observed that the highest accuracy is obtained with the green channel, both with FLANN and BF matching. The results obtained with BF matching are better, which is due to the fact that this method performs an extensive search of all possible matching points, which is not performed with FLANN to improve its computational efficiency.

**Figure 8:**
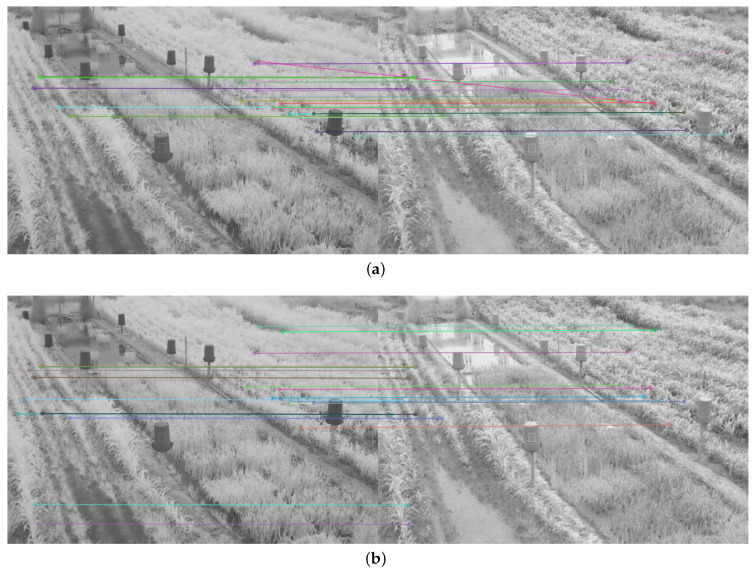
Matching results of a pair of images from the green and NIR bands of the dataset using BRISK. (**a**) FLANN matching; (**b**) BF matching.

**Figure 9:**
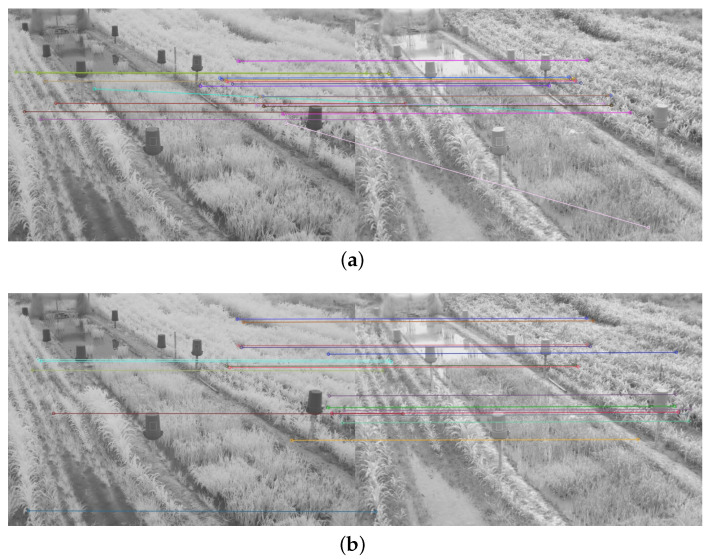
Matching results of a pair of images from the green and NIR bands image of the dataset using FAST + BRISK. (**a**) FLANN matching; (**b**) BF matching.

**Figure 10:**
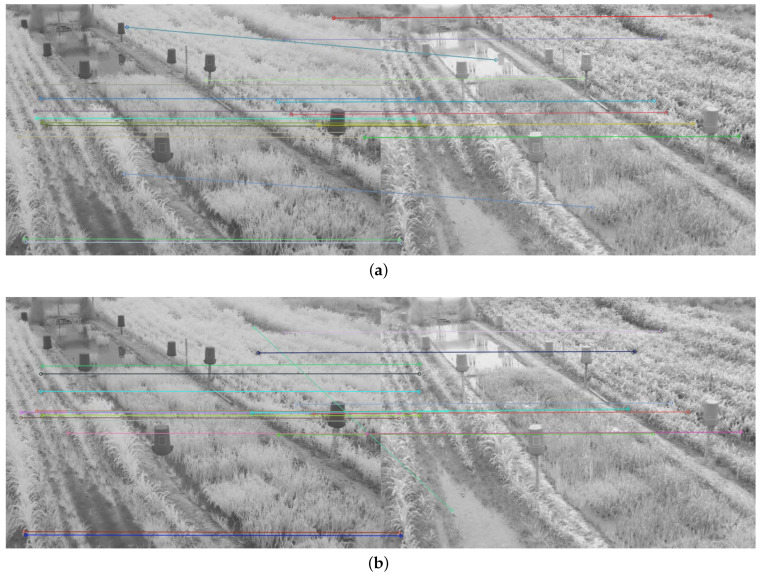
Matching results of a pair of images from the green and NIR bands image of the dataset using SURF. (**a**) FLANN matching; (**b**) BF matching.

**Figure 11:**
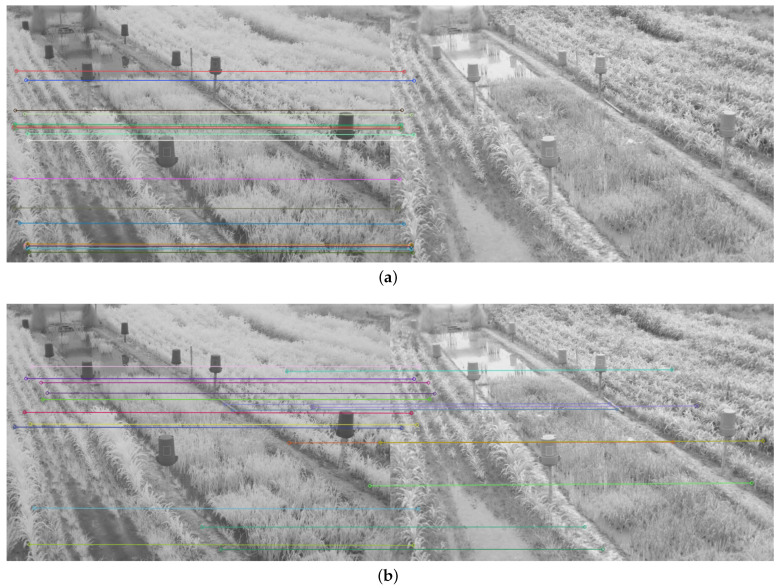
Matching results of a pair of images from the green and NIR bands image of the dataset using SIFT. (**a**) FLANN matching; (**b**) BF matching.

**Figure 12:**
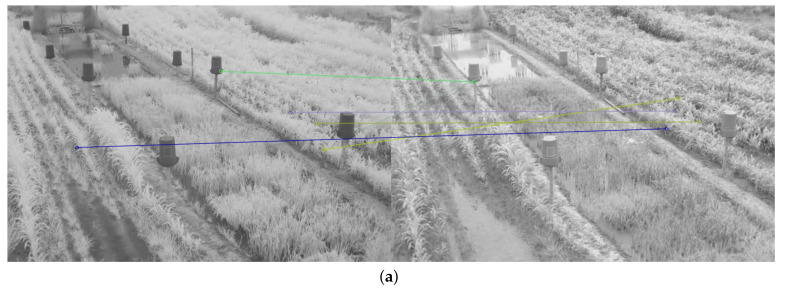

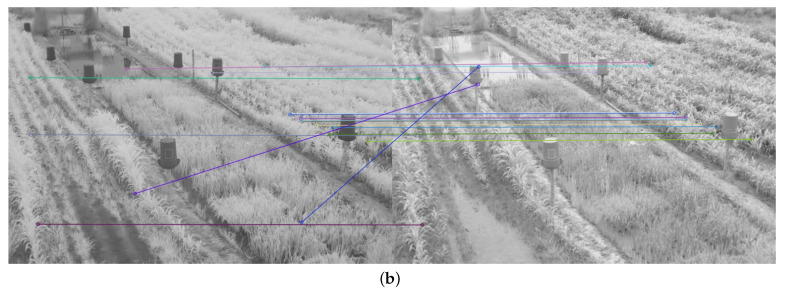
Matching results of a pair of images from the green and NIR bands image of the dataset using ORB. (**a**) FLANN matching; (**b**) BF matching.

**Figure 13:**
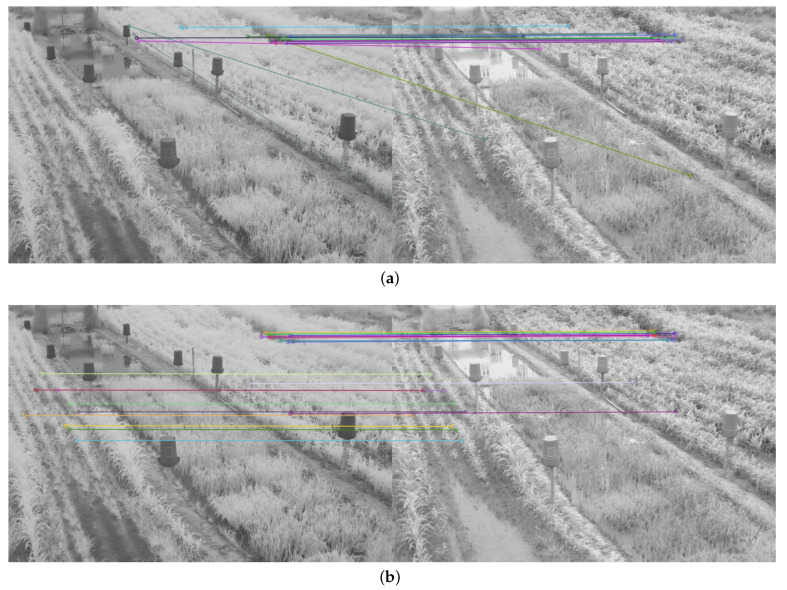
Matching results of a pair of images from the green and NIR bands image of the dataset using FAST + BRIEF. (**a**) FLANN matching; (**b**) BF matching.

**Figure 14:**
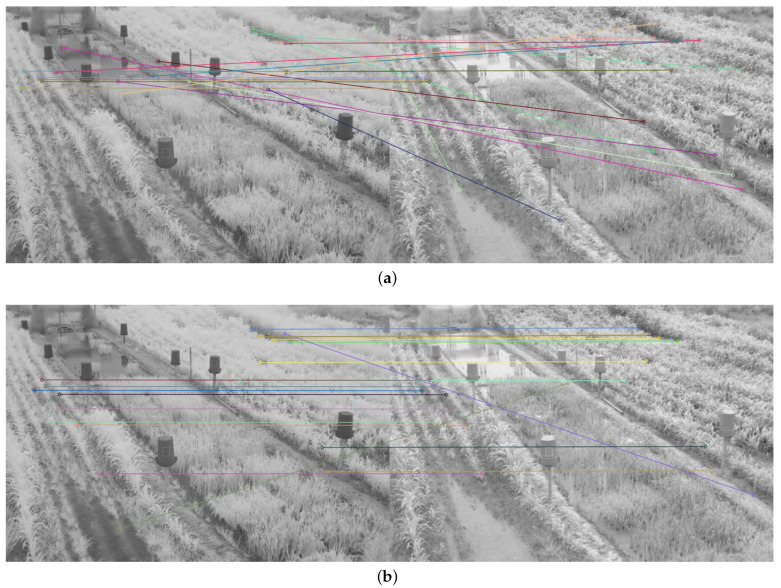
Matching results of a pair of images from the green and NIR bands image of the dataset using FAST + FREAK. (**a**) FLANN matching; (**b**) BF matching.

**Figure 15:**
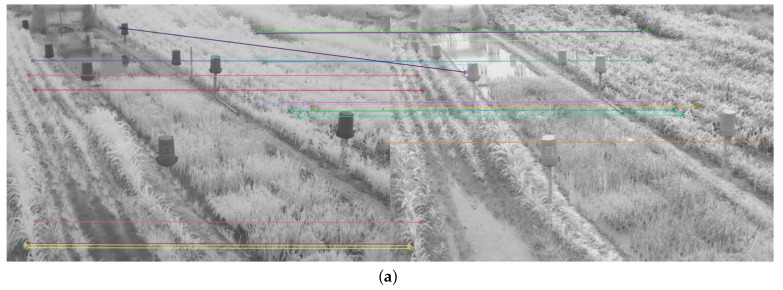

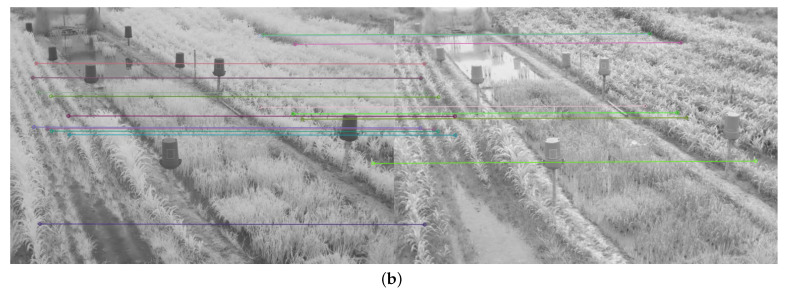
Matching results of a pair of images from the green and NIR bands image of the dataset using KAZE. (**a**) FLANN matching; (**b**) BF matching.

**Figure 16:**
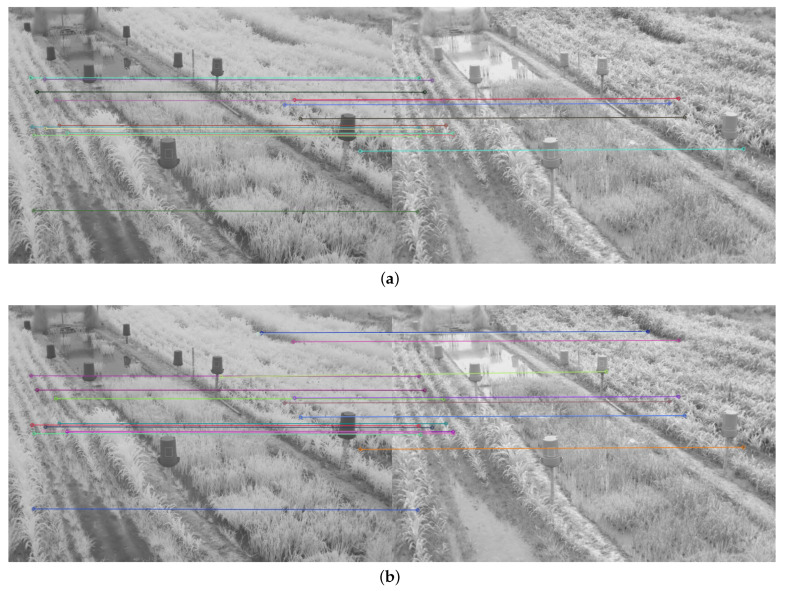
Matching results of a pair of images from the green and NIR bands image of the dataset using AKAZE. (**a**) FLANN matching; (**b**) BF matching.

Therefore, in addition to the average match results shown in Table 2 and Table 3, a display limited to 20 matches, for each feature descriptor and detector combination used, is provided in Figure 8, Figure 9, Figure 10, Figure 11, Figure 12, Figure 13, Figure 14, Figure 15 and Figure 16. A pair of images from the green and NIR bands was selected from the data set to facilitate analysis of the results.

Since the green channel showed the best performance, in terms of correct matches, for most of the feature detectors and descriptors, the matching methods exhibited similar results. The major differences could be seen using ORB (see Figure 12), FAST + BRIEF (see Figure 13) and FAST + FREAK (see Figure 14). For both ORB, FAST + BRIEF, and FAST + FREAK, BF was the most accurate matching method.

In addition, some differences can be seen in the results for other methods, like BRISK (see Figure 8), FAST + BRISK (see Figure 9), SURF (see Figure 10), and KAZE (see Figure 15). For example, BRISK and FAST + BRISK use the same descriptor, but the match points with BRISK method are more targeted. SURF results only show match points in the upper half of the images, while KAZE match points can also be seen in the lower half of the images.

In all cases, the performance is slightly better using the BF method, because fewer matching errors were made. Finally, almost no differences are observed in the cases of SIFT (see Figure 11) and AKAZE (see Figure 16). Although the exhibited performance was not always observed in all images, it is a pattern followed by most of them, as can be seen in Table 2 and Table 3.

**Figure A1:**
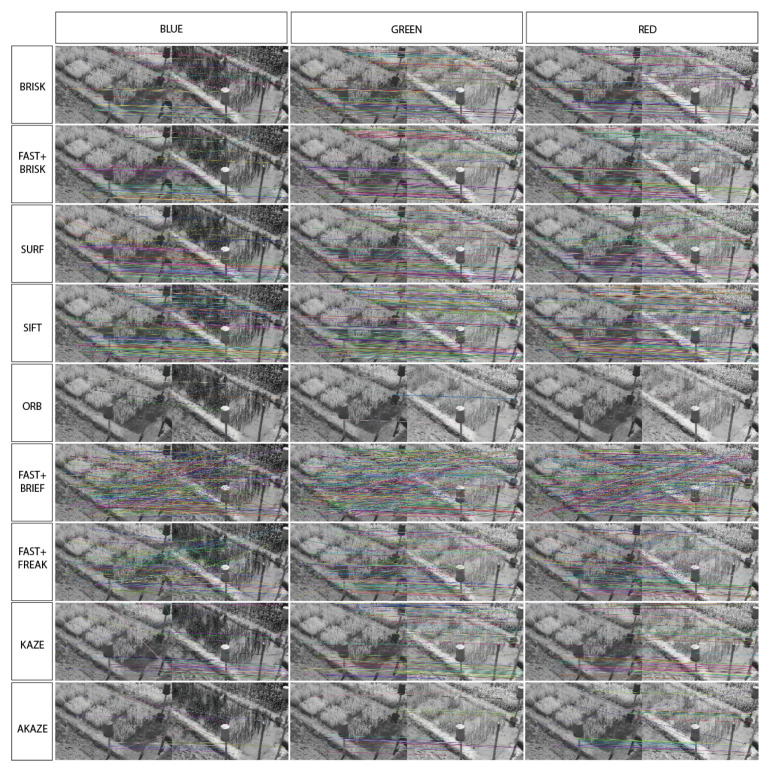
Feature matching results with all detectors and descriptors in RGB images using FLANN matching.

**Figure A2:**
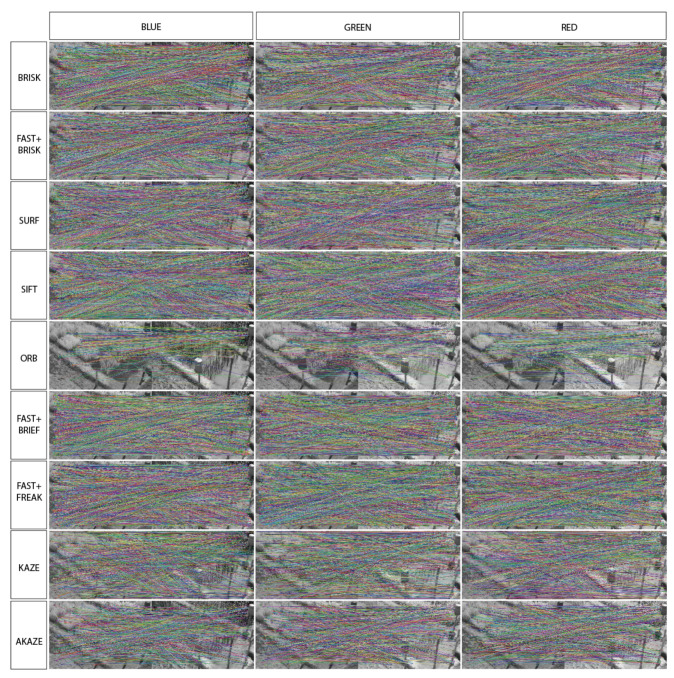
Feature matching results with all detectors and descriptors in RGB images using BF matching.

**Figure A3:**
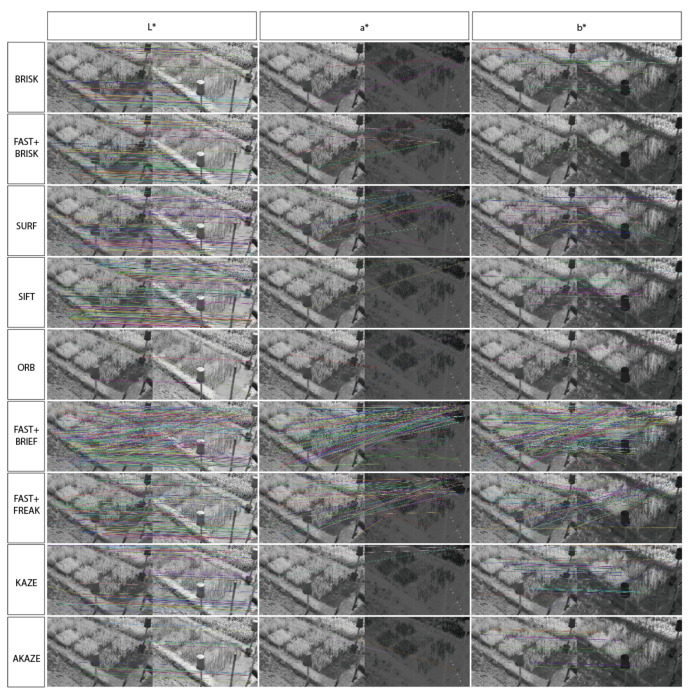
Feature matching results with all detectors and descriptors in CIE L*a*b* images using FLANN matching.

**Figure A4:**
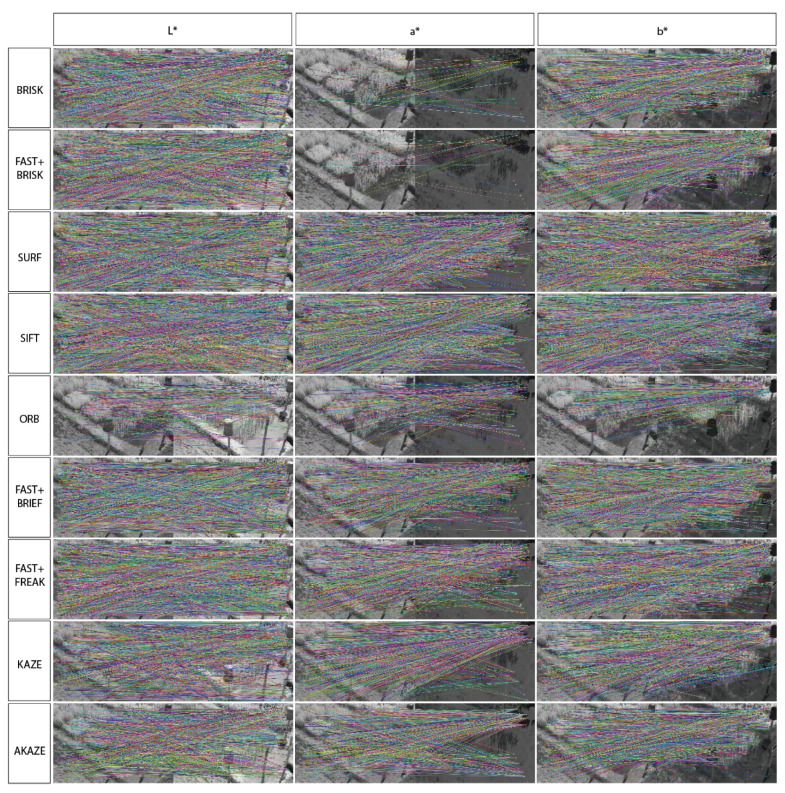
Feature matching results with all detectors and descriptors in CIE L*a*b* images using BF matching.

Moreover, from Figure 8, Figure 9, Figure 10, Figure 11, Figure 12, Figure 13, Figure 14, Figure 15 and Figure 16, it can be concluded that the matching points were mostly found in the small plants. On the other hand, only a few matching points were found in the center of the images, where the taller plants were located. This result may be due to the higher local contrast in the regions where the smaller plants and the ground are located in the NIR and VIS images. In contrast, the intensity of the tall rice displayed no significant variation. Similarly, Figure A1 and Figure A3 show that changes in pixel intensity are detectable in soil rocks. The matching results of the RGB and L* channel images exhibited this behavior. Meanwhile, the coincidences of the b* channel images in Figure A3 occurred mainly in the more developed plants.

Based on the Table 2 and Table 3, the ORB feature descriptor had fewer matching features with the FLANN and BF methods. According to Forero et al. [11] ORB method showed poor performance for pasture crop applications. In comparison, SURF and SIFT showed similar results, and matched most of the features between all RGB and CIE L*a*b* channel images and NIR images, which could be caused by the similar principles on which they are based. Noble [13] found the best results for NIR and RGB image fusion with the SURF and SIFT methods using FLANN. However, for the other techniques BF gave better results.

The results of this work allowed determining the best descriptors and matching methods to fuse RGB and NIR images of rice crops of different phenotypes, planted in small parcels, in order to derive different vegetation indices to monitor the health and productivity of rice crops. These methods can also be extended to any crop and other low-cost phenotyping platforms to obtain better crop information and select resource-efficient genotypes.

## 4. Conclusions

Image processing techniques for precision agriculture have shown great potential for applications, such as crop monitoring. Since different types of sensors and multiple feature extraction methods can be used, a comparative analysis of various feature descriptors and detectors using NIR and VIS images of rice crops was performed in this work. The results showed that the fastest algorithm was the ORB implementation, but it also had the worst performance in terms of feature matching. SIFT and FAST showed the best matches using FLANN and BF matching, respectively, on green channel images. In turn, the method that presented the highest performance in both time and number of correct matches was the combination of the FAST feature detector and the BRISK descriptor. Therefore, the various results obtained justify the need to analyze different descriptors and feature detectors to find the best performance in NIR and VIS images of rice crops. As future work, it is intended to expand the database, compare other available descriptors and detectors and apply different matching methods. Finally, possible combinations of detectors and descriptors should be tested to study their performance depending on the used database.

The RGB images will bring good spatial resolution to monitor the crops at different scales, but it attains more information than RGB alone when it merges with NIR images. The methods or techniques developed from this paper will be helpful to the researchers to combine RGB and NIR images to estimate several vegetation indices to monitor crop health and productivity at different time points. Unfortunately, the hi-tech multispectral cameras are expensive, and its expensive need drones to monitor the larger areas. Still, when the experimental site is small, it is unnecessary to use the drone. Instead, cheaper RGB cameras with NIR filters can be attached to capture multiple field images and then can be merged. These low-cost cameras can be attached to the towers or moving ground vehicles to capture many high-resolution RGB-NIR images around the plots can be easily integrated using advanced fusion techniques. In this paper, we used small plot rice RGB and NIR images collected from phenotowers around the field, demonstrating efficient methods to fuse images accurately and quickly. The merging techniques employed in this research will be more beneficial to rapidly merge RGB and NIR images to derive different vegetation indices to monitor the health and productivity of rice crops. These methods can also be extended to any crop and other low-cost phenotyping platforms to obtain better crop information and select resource-efficient genotypes. 

## Figures and Tables

**Table 1:** BF matching distance for feature descriptors.

Feature Descriptor	Matching Distance	OpenCV Function
SURF	L1 norm	NORM_L1
SIFT		
BRISK		
KAZE		
AKAZE		
ORB	Hamming norm	NORM_HAMMING2
BRISK	Hamming norm	NORM_HAMMING
FAST + BRIEF		
FAST + BRISK		
FAST + FREAK		

**Table 2:** Average matches of feature descriptors and detectors evaluated on each channel of RGB and CIE L*a*b* dataset images using FLANN matching method. The best values for each method are shown in bold.

		FLANN Matching								
**Images**		**BRISK**	**FAST + BRISK**	**SURF**	**SIFT**	**ORB**	**FAST + BRIEF**	**FAST + FREAK**	**KAZE**	**AKAZE**
RGB	Blue	6.00	8.00	9.45	13.00	0.70	4.55	3.90	4.50	2.55
	Red	10.25	12.45	12.20	16.80	1.30	3.40	3.50	9.30	5.05
	Green	**13.85**	**13.90**	13.90	**17.40**	1.35	**6.45**	**6.75**	12.00	6.70
CIE L*a*b	L*	11.55	13.70	**14.00**	17.00	1.20	6.00	6.15	11.45	6.10
	a*	0.20	0.05	0.50	0.20	0.50	0.00	0.00	0.25	0.05
	b*	6.20	0.35	10.90	12.70	**2.45**	0.00	0.00	**14.60**	**12.50**

**Table 3:** Average matches of feature descriptors and detectors evaluated on each channel of RGB and CIE L*a*b* dataset images using BFe matching method. The best values for each method are shown in bold.

		BF Matching								
**Images**		**BRISK**	**FAST + BRISK**	**SURF**	**SIFT**	**ORB**	**FAST + BRIEF**	**FAST + FREAK**	**KAZE**	**AKAZE**
RGB	Blue	7.80	9.35	11.30	11.70	2.70	14.40	13.60	5.65	4.30
	Red	14.05	15.15	14.80	15.30	6.25	9.50	10.20	12.15	8.10
	Green	**16.90**	**17.25**	**16.60**	16.50	8.55	**17.00**	**16.20**	**13.65**	10.05
CIE L*a*b*	L*	11.55	16.15	16.50	**16.60**	6.60	15.50	14.00	12.90	9.55
	a*	0.20	0.00	0.45	0.55	0.40	0.05	0.10	0.75	0.45
	b*	6.20	1.60	9.45	9.05	**11.20**	0.20	0.10	17.10	**16.75**

**Table 4:** Execution time of the evaluated feature descriptors and detectors in RGB and CIE L*a*b* dataset images using FLANN and BF matching. The best values for each method are shown in bold.

Time (s)										
**Matching** **Method**	**Image** **Channel**	**BRISK**	**FAST+** **BRISK**	**SURF**	**SIFT**	**ORB**	**FAST+** **BRIEF**	**FAST+** **FREAK**	**KAZE**	**AKAZE**
FLANN	Blue	0.567	0.469	0.537	0.535	**0.054**	0.472	0.842	**1.133**	0.231
	Green	0.552	0.461	0.546	0.530	**0.052**	0.469	0.844	**1.152**	0.229
	Red	0.556	0.459	0.559	0.533	**0.052**	0.460	0.843	**1.096**	0.220
	L*	0.489	0.377	0.586	0.547	**0.055**	0.368	0.652	**1.113**	0.228
	a*	0.340	0.273	0.471	0.377	**0.050**	0.196	0.368	**1.107**	0.227
	b*	0.374	0.299	0.502	0.428	**0.051**	0.239	0.387	**1.160**	0.241
Brute Force	Blue	0.596	0.438	0.822	1.028	**0.050**	1.303	**1.781**	**1.298**	0.215
	Green	0.452	0.409	0.727	0.909	**0.049**	1.043	1.250	1.207	0.194
	Red	0.437	0.367	0.651	0.850	**0.038**	0.997	**1.503**	1.133	0.218
	L*	0.824	0.654	1.326	**1.676**	**0.062**	**1.870**	**2.575**	1.397	0.275
	a*	0.471	0.395	0.830	0.624	**0.049**	0.422	0.651	1.382	0.262
	b*	0.487	0.384	0.980	0.865	**0.051**	0.570	0.795	1.401	0.269

## Data Availability

The data used to support the findings of this study are available from the corresponding author upon request.

## References

[1] Tang Y., Dananjayan S., Hou C., Guo Q., Luo S., He Y. (2021). A survey on the 5G network and its impact on agriculture: Challenges and opportunities. Comput. Electron. Agric..

[2] Barzin R., Pathak R., Lotfi H., Varco J., Bora G.C. (2020). Use of UAS Multispectral Imagery at Different Physiological Stages for Yield Prediction and Input Resource Optimization in Corn. Remote Sens..

[3] Saleem S., Bais A., Sablatnig R. (2016). Towards feature points based image matching between satellite imagery and aerial photographs of agriculture land. Comput. Electron. Agric..

[4] Russello H. (2018). Convolutional Neural Networks for Crop Yield Prediction. Ph.D. Thesis.

[5] Habib A., Han Y., Xiong W., He F., Zhang Z., Crawford M. (2016). Automated ortho-rectification of UAV-based hyperspectral data over an agricultural field using frame RGB imagery. Remote Sens..

[6] Rondao D., Aouf N., Richardson M.A., Dubois-Matra O. (2020). Benchmarking of local feature detectors and descriptors for multispectral relative navigation in space. Acta Astronaut..

[7] Yan J., Liu Y. A Stereo Visual Obstacle Detection Approach Using Fuzzy Logic and Neural Network in Agriculture. Proceedings of the 2018 IEEE International Conference on Robotics and Biomimetics, ROBIO 2018.

[8] Chen S., Yuan X., Yuan W., Niu J., Xu F., Zhang Y. (2018). Matching Multi-Sensor Remote Sensing Images via an Affinity Tensor. Remote Sens..

[9] Jiang J., Feng X., Liu F., Xu Y., Huang H. (2019). Multi-Spectral RGB-NIR Image Classification Using Double-Channel CNN. IEEE Access.

[10] Yang Q., Shi L., Han J., Zha Y., Zhu P. (2019). Deep convolutional neural networks for rice grain yield estimation at the ripening stage using UAV-based remotely sensed images. Field Crop. Res..

[11] Forero M.G., Acosta A., Ducuara A.S., Godoy Y.A., Moreno J.G. Comparative Analysis of Interest Points Detection Techniques for the Registration of Infrared and Visible Aerial Photographs in Pasture Crops. Proceedings of the SPIE 11137, Applications of Digital Image Processing XLII, 111370G.

[12] Haghverdi A., Washington-Allen R.A., Leib B.G. (2018). Prediction of cotton lint yield from phenology of crop indices using artificial neural networks. Comput. Electron. Agric..

[13] Noble F.K. Comparison of OpenCV’s feature detectors and feature matchers. Proceedings of the 2016 23rd International Conference on Mechatronics and Machine Vision in Practice (M2VIP).

[14] Cowan B., Imanberdiyev N., Fu C., Dong Y., Kayacan E. A performance evaluation of detectors and descriptors for UAV visual tracking. Proceedings of the 2016 14th International Conference on Control, Automation, Robotics and Vision (ICARCV).

[15] Jafari Malekabadi A., Khojastehpour M., Emadi B. (2018). A comparative evaluation of combined feature detectors and descriptors in different color spaces for stereo image matching of tree. Sci. Hortic..

[16] Fairchild M.D. (2019). CIE 015:2018 Colorimetry.

[17] Lowe D.G. Object Recognition from Local Scale-Invariant Features. Proceedings of the IEEE International Conference on Computer Vision.

[18] Bay H., Tuytelaars T., Gool L.V. LNCS 3951—SURF: Speeded Up Robust Features. Proceedings of the 9th European Conference on Computer Vision (ECCV 2006).

[19] Rosten E., Drummond T., Leonardis A., Bischof H., Pinz A. (2006). Machine learning for high-speed corner detection. Computer Vision—ECCV 2006.

[20] Leutenegger S., Chli M., Siegwart R.Y. BRISK: Binary Robust invariant scalable keypoints. Proceedings of the IEEE International Conference on Computer Vision.

[21] Calonder M., Lepetit V., Strecha C., Fua P., Daniilidis K., Maragos P., Paragios N. (2010). BRIEF: Binary Robust Independent Elementary Features. Computer Vision—ECCV 2010.

[22] Rublee E., Rabaud V., Konolige K., Bradski G. ORB: An efficient alternative to SIFT or SURF. Proceedings of the IEEE International Conference on Computer Vision.

[23] Alahi A., Ortiz R., Vandergheynst P. FREAK: Fast Retina Keypoint. Proceedings of the 2012 IEEE Conference on Computer Vision and Pattern Recognition.

[24] Alcantarilla P.F., Bartoli A., Davison A.J. (2012). KAZE features. Lect. Notes Comput. Sci..

[25] Alcantarilla P.F., Nuevo J., Bartoli A. Fast explicit diffusion for accelerated features in nonlinear scale spaces. Proceedings of the BMVC 2013—Electronic Proceedings of the British Machine Vision Conference 2013.

[26] Schmittmann O., Lammers P.S. (2017). A True-Color Sensor and Suitable Evaluation Algorithm for Plant Recognition. Sensors.

